# Left Ventricular Dysfunction Caused by Unrecognized Surgical AV block in a Patient with a Manifest Right Free Wall Accessory Pathway

**DOI:** 10.1016/s0972-6292(16)30627-1

**Published:** 2013-06-25

**Authors:** Rakesh Gopinathannair, Dwayne N Campbell, Alexander Mazur

**Affiliations:** 1 Division of Cardiovascular Medicine, Section of Electrophysiology, University of Louisville, Louisville, KY; 2 Division of Cardiovascular Medicine, Section of Electrophysiology, University of Iowa Hospitals, Iowa City, IA

**Keywords:** Left ventricular dysfunction, accessory pathway, right free wall, complete AV block, dyssynchrony

## Abstract

A 24-year-old male with Wolff-Parkinson-White syndrome developed systolic cardiomyopathy and severe heart failure following membranous ventricular septal defect repair and tricuspid valve replacement. Following successful catheter ablation of a right anterolateral accessory pathway (AP), complete AV block with junctional escape rhythm was noted. Patient subsequently underwent implantation of a biventricular ICD. Heart failure symptoms significantly improved soon after and left ventricular systolic function normalized 3 months post-procedure. In this case, surgically acquired AV block likely explains development of postoperative cardiomyopathy by facilitating ventricular activation solely via the AP and thereby increasing the degree of ventricular dyssynchrony.

## Case Report

A 24-year-old male with history of congenital membranous ventricular septal defect (VSD) developed tricuspid valve endocarditis following a dental procedure in 2007 and subsequently underwent VSD closure and bioprosthetic tricuspid valve replacement. His left ventricular (LV) ejection fraction prior to surgery was normal. In 2009, he presented with heart failure symptoms and was found to have severe LV systolic dysfunction. Since LV dysfunction persisted despite optimal medical therapy, he was referred to electrophysiology for consideration of a primary prevention ICD in 2010.

**Figure 1:**
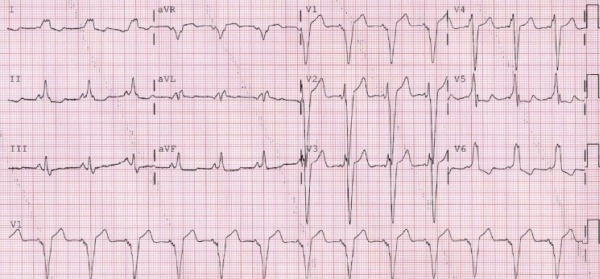
Baseline 12-lead electrocardiogram at current admission showing sinus rhythm with ventricular pre-excitation. Note prolonged QRS with duration of 193 msec. Left bundle branch morphology in lead V1 with late precordial transition and positive delta waves in inferior leads are consistent with a right anterolateral accessory pathway.

His 12-lead electrocardiogram at baseline (Figure 1) showed sinus rhythm with a QRS pattern consistent with ventricular pre-excitation over a right free wall accessory pathway (AP). The QRS duration was 193 msec. A transthoracic echocardiogram showed normal LV size with evidence of abnormal septal wall motion and an LV ejection fraction of 35%. The bioprosthetic tricuspid valve showed normal function and there was no evidence of residual VSD. A cardiac CT revealed normal coronary arteries and a cardiac MRI showed no evidence of delayed enhancement. We felt that the ventricular preexcitation pattern mimicking left bundle branch block could potentially contribute to his cardiomyopathy by causing dyssynchrony [1,2]. Therefore, the patient underwent electrophysiology study with a plan for catheter ablation of the AP.

**Figure 2:**
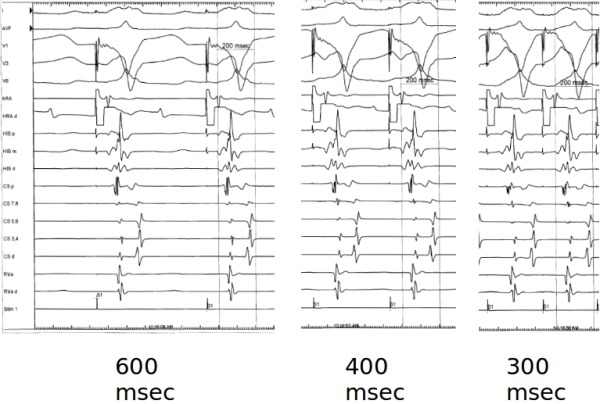
No change in QRS duration or ventricular activation sequence noted during atrial pacing (from high right atrium) at different cycle lengths.

Baseline rhythm was sinus with a preexcited QRS complex of 193 msec. The HV interval was negative. The antegrade and retrograde AP block cycle length were 270 and 280 msec, respectively. Retrograde atrial activation was entirely eccentric with the earliest local activation in the area of the antero-lateral tricuspid annulus. The antegrade AP effective refractory period (ERP) was 320/600 msec. No significant changes in QRS duration or pattern were noted during atrial programmed stimulation and decremental pacing (Figure 2). The retrograde AP ERP was ≤ ventricular ERP (240/600 msec). No arrhythmia could be induced with aggressive atrial and ventricular stimulation protocol (with or without isoproterenol).

Mapping along the atrial aspect of the tricuspid annulus was performed using a 4 mm solid tip ablation catheter (St. Jude Medical, Minneapolis, MN). This revealed earliest endocardial ventricular and atrial activation to be at the anterolateral tricuspid annulus. A pre-formed sheath (SR2, St. Jude Medical, Minneapolis, MN) was employed to improve catheter stability in this area. During mapping, mechanical block of pathway conduction was noted. RF lesions deployed at this location resulted in permanent abolition of accessory pathway conduction.

**Figure 3:**
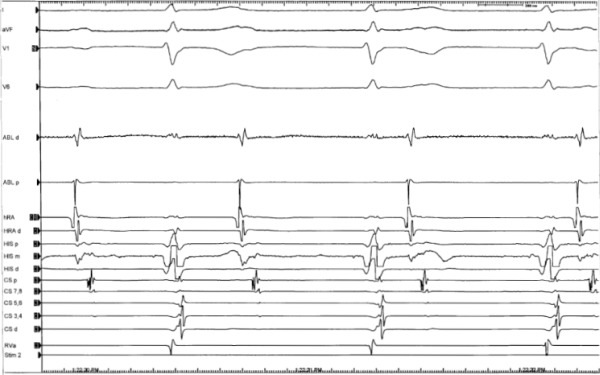
Complete heart block with a junctional escape rhythm seen following mechanical block ("bump map") of the accessory pathway. Distal ablation catheter (ABLd) shows local electrograms at the site of mechanical block. Ablation at this location eliminated pathway conduction.

Post-ablation, complete AV block with a junctional escape rhythm of 60-65 beats/min was noted (Figure 3). Damage to the AV node probably occurred during prior cardiac surgery and was masked by antegrade preexcitation until now.

**Figure 4:**
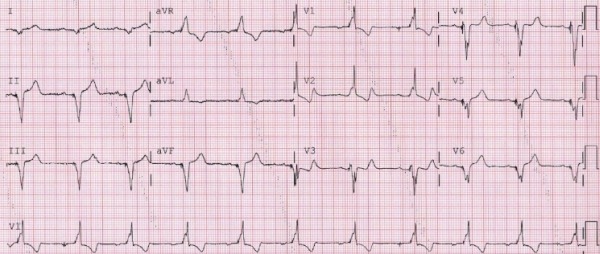
Twelve-lead electrocardiogram during biventricular pacing.

In view of significant LV dysfunction with heart failure symptoms and complete AV block, the patient underwent implantation of a biventricular ICD the following day. A 12-lead electrocardiogram showing sinus rhythm with biventricular pacing is shown in Figure 4. At a 2-week follow up visit, significant improvement in heart failure symptoms and exercise tolerance was seen. The underlying intrinsic rhythm was complete AV block. Serial echocardiograms done at 3-month and 1-year follow up visits showed complete normalization of LV systolic function and wall motion with an estimated LV ejection fraction of 55%.

## Discussion

A small percentage of patients with WPW syndrome have been reported to develop dilated cardiomyopathy in the absence of AP-mediated tachyarrhythmias [1,3,4]. Retrospective evidence suggests a strong association between right-sided septal or paraseptal AP location and development of LV dysfunction [1,5]. Potential mechanistic relation between ventricular preexcitation and LV systolic dysfunction is further supported by the fact that successful ablation of AP can reverse LV dysfunction [1,3,6].

The exact mechanism leading to cardiomyopathy in patients with ventricular preexcitation is not completely understood. A potential explanation is that premature ventricular activation over a right-sided pathway causes abnormal septal wall motion similar to that seen in left bundle branch block or right ventricular apical pacing [1,2,4,5]. The resultant ventricular dyssynchrony is responsible for adverse LV remodeling and LV systolic dysfunction [1,2,4,5]. The time required to develop cardiomyopathy and the precipitating factors remain unknown.

Our patient had normal LV dimensions and ejection fraction prior to VSD repair and tricuspid valve replacement in 2007. Extensive testing excluded common etiologies for cardiomyopathy and the somewhat rapid deterioration in LV function appeared puzzling. Although residual myocardial damage from VSD, endocarditis, intra-operative ischemia or cardioplegia can cause postoperative cardiomyopathy, complete reversal of LV systolic function by AP ablation and biventricular ICD placement practically rules out these mechanisms. Underlying complete AV block also rules out undetected AP-mediated tachyarrhythmias as a potential cause of his cardiomyopathy.

**Figure 5:**
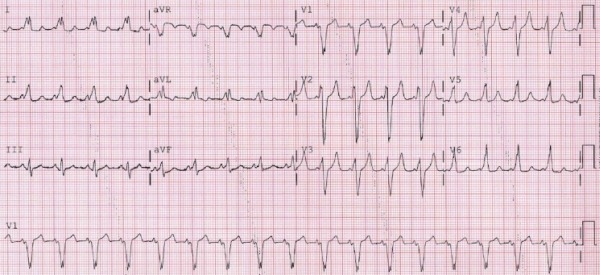
Twelve-lead electrocardiogram obtained 4 months before patient underwent VSD repair and tricuspid valve replacement. This shows sinus rhythm with ventricular pre-excitation consistent with a right anterolateral accessory pathway. The QRS duration is 157 msec.

Once the AP was ablated successfully, sinus rhythm with complete AV block was noted. Although damage to the AV node during AP ablation is theoretically possible, this is highly unlikely in this AP location. Moreover, no change in QRS duration/pattern with decremental atrial pacing and no evidence of retrograde AV nodal conduction in a young patient prior to ablation strongly argue against this possibility. We postulate that irreversible AV node damage occurred during the VSD repair and tricuspid valve surgery. This could have resulted in antegrade conduction being entirely through the AP, thus significantly increasing the degree of septal dyssynchrony and facilitating development of cardiomyopathy. 12-lead electrocardiogram comparison prior to (Figure 5) and after cardiac surgery in 2007 (Figure 1) demonstrating significant increase in QRS duration (from 157 msec to 193 msec), likely secondary to loss of antegrade AV nodal conduction, provides support to this hypothesis. Thus, the presence of good AV node conduction, by allowing fusion of the conducted sinus wavefront, may act as a "buffer", mitigating the degree of ventricular preexcitation over an AP, and thereby protecting against the development of LV dyssynchrony and adverse remodeling. To our knowledge, this is the first report providing insight into a potential mechanism that can precipitate development of LV dysfunction in the presence of a right-sided AP. This case suggests that preservation of AV nodal conduction may play an important role in preventing cardiomyopathy. Further studies are needed to confirm this hypothesis.

Underlying complete AV block and the presence of significant LV dysfunction necessitated biventricular pacing, in addition to AP ablation, for reversal of LV dysfunction in our case. Although one cannot clearly quantify the added benefit of biventricular pacing in this case, it seemed the most appropriate therapy given the presence of complete AV block post-ablation necessitating ventricular pacing. Pacing the right ventricle only in this case, by continuing to maintain a left bundle branch block QRS pattern, would have resulted in persistent ventricular dyssynchrony, the likely primary mechanism of cardiomyopathy in this patient.
